# Learning or Boredom? Task Adaptation Effects in Sentence Processing Experiments

**DOI:** 10.1162/opmi_a_00173

**Published:** 2024-12-15

**Authors:** Jan Chromý, Fabian Tomaschek

**Affiliations:** Institute of Czech Language and Theory of Communication, Faculty of Arts, Charles University, Prague, Czechia; University of Bern, Bern, Switzerland

**Keywords:** task adaptation, sentence processing, information recall, comprehension, Czech

## Abstract

Task adaptation, characterized by a progressive increase in speed throughout experimental trials, has been extensively observed across various paradigms. Yet, the underlying mechanisms driving this phenomenon remain unclear. According to the learning-based explanation, participants are implicitly learning, becoming more proficient over time. Conversely, a motivation-based view suggests that participants’ drive wanes gradually, prompting quicker pace and reduced task engagement. These explanations offer distinct predictions. The learning-based view anticipates not only accelerated speed but also improved response accuracy. In contrast, the motivation-based view assumes that participants lose their focus, their pace increases, but their response accuracy tends to decline. The present study tests these implications in a series of six self-paced reading experiments investigating the interplay between reaction times, immediate recall, and trial order. Robust learning effects are documented. Participants not only read progressively faster during the experiments, but they also get better in responding. Moreover, an analysis of recall accuracy reveals systematic differences between different types of information, with nouns yielding substantially higher recall accuracy than adjectives. These findings are explained through attentional mechanisms: prolonged processing of specific words correlates with improved recall. Furthermore, the differential recall patterns are modulated by the task’s question structure, with adjectives recalled more effectively in experiments with a higher proportion of adjective-targeting questions. This underscores participants’ strategic allocation of attention to sentence components deemed crucial for task performance, highlighting the dynamic interplay between learning, motivation, and attentional mechanisms in task adaptation.

## INTRODUCTION

Imagine being a fresh undergraduate student offered an opportunity to earn course credits through a sentence processing experiment. Your task is simple: read around 100 sentences word by word by pressing a button to reveal each word and hide the previous one. After each sentence, you also have to answer a comprehension question. At first, you take the experiment seriously, and your progress is a bit slow due to unfamiliarity. However, as you get used to it, your reading speed improves. After reading dozens of sentences, you become completely accustomed to the task, which may even lead to occasional boredom. The repetitive button pressing starts to lose its appeal, and you eagerly look forward to the experiment’s end.

Such a scenario is far from rare. Extensive evidence demonstrates gradual acceleration of participants’ pace throughout experimental tasks, influenced by their increasing experience with the task (e.g., Wells et al., 2009). This phenomenon has been coined “task adaptation” and has been observed in various experimental paradigms, including the lexical decision task (Gagné & Spalding, 2014), structural priming (Fine & Jaeger, 2013), word restoration (Arehalli & Wittenberg, 2021), self-paced reading (Fine et al., 2013; Prasad & Linzen, 2021), eye-tracking (Yan & Jaeger, 2020), and the sentence completion task (Dempsey et al., 2024). Moreover, there is also neurophysiological evidence for adaptation effects (e.g., Hahne & Friederici, 1999; Noppeney & Price, 2004).

Crucially, two different accounts have been proposed to explain the observed increase in processing speed. According to the learning-based view, participants are implicitly learning the requirements of the task, gradually refining their proficiency to perform the task as they progress through the experiment (Hodapp & Rabovsky, 2024; Tomaschek et al., 2024). As Ramscar and Baayen (2013) put it, learning is a “process that reapportions attentional/representational resources in order to maximize future predictive success”. This can be understood as a process of updating expectations both about the task itself and about the characteristics of presented stimuli (e.g., their syntactic patterns, plausibility, length, types of comprehension questions asked etc.). Conversely, a motivation-based view suggests a gradual decline in participants’ motivation, with their primary goal being to complete the experiment as swiftly as possible (cf. Christianson et al., 2022). While these explanations need not be mutually exclusive and may coexist in certain instances, they differ significantly in their implications. The learning-based view predicts not only an improvement in speed but also an increase in accuracy regarding comprehension questions, reflecting participants’ learning process. In contrast, the motivation-based account assumes a scenario where growing disinterest or boredom leads to faster processing speed but does not necessarily enhance response accuracy; in fact, it may even lead to a decline.

In the domain of sentence comprehension, learning-based assupmtions have been examined in relation to the idea of syntactic adaptation (Fine et al., 2013; Ryskin et al., 2017; Wells et al., 2009; Yan & Jaeger, 2020). According to this account, participants gradually accustom to syntactically unusual (e.g., ambiguous) or even ungramatical sentences (e.g., *While Anna dressed the baby that was cute played on the bed*) throughout the course of an experiment, leading to a reduction in measured effects such as reaction times and fixation times. However, Prasad and Linzen (2021) have recently cast doubt on the existence of syntactic adaptation effects, suggesting that these effects may largely stem from the general increase in processing speed observed during the experiment. Moreover, studies on syntactic adaptation have typically focused solely on the online aspects of syntactic adaptation, i.e., changes in magnitude of reaction time effects. This is problematic, because, as Christianson et al. (2022) put it, such measures do not offer a clear-cut interpretation (see also Ferreira & Yang, 2019, for a thorough discussion of this issue).

Importantly, two studies have attempted to examine syntactic adaptation from both the perspectives of online processing and comprehension accuracy (Dempsey et al., 2020, 2024). Interestingly, these studies have yielded somewhat divergent findings. While Dempsey et al. (2020) found no evidence of adaptation in terms of comprehension accuracy, measured through comprehension questions, Dempsey et al. (2024) reported an improvement in this measurement for garden-path structures, i.e., sentences containing local ambiguity, such as *The vendor sold the hotdog and the popcorn was still stale*.

A serious confound in previous studies on task (or syntactic) adaptation arises from the inclusion of control items. There is ample evidence that learning is a continuously ongoing process that happens everytime one performs any kind of action (see Heitmeier et al., 2023, for effects in semantics), even when the perceived input is seemingly random (Tomaschek et al., 2024). This means that, from a learning perspective, one would expect that each sentence and comprehension question used in the experiment alters expectations, predictions, or even attention to specific segments of the sentence (e.g., Swets et al., 2008). While repeated exposure to a garden-path sentence, for instance, may lead to faster and more accurate processing of such locally ambiguous sentences, it is also plausible that exposure to control, non-garden-path sentences prompts “unlearning”, i.e., reduced expectation of locally ambiguous sentences due to the presence of unambiguous ones.

In sum, the evidence in favour of gradual acceleration in the processing speed throughout an experiment is overwhelming, yet the explanation for this phenomenon remains elusive. Distinguishing between the learning-based and motivation-based speed-up remains challenging. Nonetheless, understanding these underlying mechanisms is crucial for accurately interpreting and generalizing the findings of sentence processing experiments. Shedding light on the interplay between learning and motivation can enhance our understanding of how participants adapt to experimental tasks and contribute to a more nuanced comprehension of human cognition.

The primary objective of this paper is to investigate task adaptation in both reaction times and comprehension accuracy. We employ the word-by-word self-paced reading task with open-ended questions, similar to the approach employed by Ozuru et al. (2013) or Ceháková and Chromý (2023). One of the main advantages of using open-ended questions in comparison to the typically used yes-no questions is the fact that participants cannot simply guess the correct answer; instead, they must actively formulate their answers without the aid of predefined options.

Notably, this study explores task adaptation effects using sentences which are unambiguous and entirely plausible. The sentences used in this study are somewhat similar to those used by Chromý and Vojvodić (2024). In this study, systematic differences in immediate recall between direct objects and locative adjuncts were documented. Direct objects were nearly always recalled correctly, while information conveyed through locative adjuncts exhibited significantly lower recall rates. The results were also influenced by information structure: in case the piece of information was focused in the sentence, its recall was higher than if it was not focused. In the present study, we thus target this distinction too. Moreover, we also target adjectives modifying direct object noun and locative adjunct noun, since we found in a previous pilot study that the recall of adjective information is rather weak which offered us a broader range of variation in which task adaptation may manifest itself clearly.

Crucially, the current study diverges from conventional methodology by avoiding the use of filler items. While this approach may seem unorthodox, it offers a distinct advantage in terms of controlling for learning/adaptation effects throughout the experiment.

## METHODS

### Ethics Approval

Research ethics board approval for this study was acquired from the Research Ethics Committee, Faculty of Arts, Charles University (Ref. No.: UKFF/624176/2023). Participation in the experiments was voluntary, and all participants provided written informed consent. All data used in the current analyses were fully anonymized.

### Participants

A total of 1472 (1182 female, 279 male, 11 not disclosed) native speakers of Czech were recruited from the participant pool of the Charles University (Prague, Czech Republic) and received course credits for their participation. The distribution of participants across experiments was: 224 in Experiment 1, 215 in Experiment 2, 201 in Experiment 3, 230 in Experiment 4, 322 in Experiment 5, 280 in Experiment 6. Each participant participated in only one of these experiments. Participants were on average 22.9 years old (*SD* = 5.6 years).

### Experiment Design and Material

The experiment was conducted online using PC Ibex Farm platform (Zehr & Schwarz, 2018). Prior to the experimental trials, a brief demographic questionnaire was administered, where participants were asked to provide their age, gender and native language, in addition to whether they experience any reading problems (such as dyslexia). In case they responded “yes” to the final question, they were not included in the analysis.

To investigate the research questions at hand, we performed a total of six online self-paced experiments. In each experiment, the participants read a total of 96 Czech sentences which differed in their word order and also in the obligatoriness of the prepositional phrase referring to the location. The word order variable had two values: (a) svopp (subject – verb – object – prepositional phrase), and (b) ppvso (prepositional phrase – verb – subject – object). Both word orders are possible in Czech and they are used for manipulating the information structure (Siewierska & Uhliřová, 1998). In svopp, the prepositional phrase (i.e., the location) is focused, whereas in ppvso, the object information is focused. The prepositional phrase obligatoriness was set based on the valency dictionary of Czech verbs Vallex 4.0 (Lopatková et al., 2020) and two values were used: (i) obligatory prepositional phrase (i.e., the sentence would not be grammatical without it), (ii) optional prepositional phrase (i.e., it is not necessary to use such a phrase to create a grammatical sentence). Each combination of values of word order and PP obligatoriness presented 24 items. The adjectives modyfing the object or prepositional phrase were always selected so that they would be in principle interchangeable, namely that the adjective modyfing the object may potentially plausibly modify the locative adjunct and vice versa. The example items are presented in Table 1.

**Table 1.** Items example from Experiments 1, 2, and 3 together with their English translations.

Word order	PP obligatoriness	1	2	3	4	5	6	7
svopp	obligatory	Anna	nacpala	obstarožní	sukni	do	béžové	skříně.
		Anna	stuffed	outmoded	skirt	into	beige	wardrobe
		‘*Anna stuffed the old-fashioned skirt into the beige wardrobe.*’						
ppvso	obligatory	Na	starý	věšák	pověsil	Denis	modrý	svetr.
		on	old	hanger	hanged	Denis	blue	sweater
		‘*Denis hanged the blue sweater on an old hanger.*’						
svopp	arbitrary	Student	četl	italský	rukopis	v	zatuchlém	archivu.
		student	read	Italian	manuscript	in	musty	archive
		‘*The student read an Italian manuscript in a musty archive.*’						
ppvso	arbitrary	Na	dlouhém	meetingu	přečetl	Daniel	důležitý	dokument.
		at	long	meeting	read	Daniel	important	document
		‘*Daniel read out an important document at an important meeting.*’						

After having read each sentence, participants had to type answers to an open-ended comprehension question targeting certain piece of information presented in the sentence. Four types of questions were used:

(a)  questions targeting direct object (e.g., ‘What did Anna stuff into the wardrobe?’)
(b)  questions targeting locative prepositional phrase (e.g., ‘Where did Anna stuff the skirt?’)
(c)  questions targeting adjectives modifying the direct object (e.g., ‘What kind of skirt was it?’)
(d)  questions targeting adjectives modifying the prepositional phrase (e.g., ‘What kind of wardrobe was it?’)

Typed answers were manually coded for response correctness, following a coding procedure similar to that used by Chromý and Vojvodić (2024). Correct answers included both verbatim repetitions of the sentence content and nonverbatim, but semantically accurate responses (e.g., synonyms), regardless of any orthographical errors. Some responses contained more information than was explicitly requested by the questions. In such cases, coding focused solely on the target information—either the noun or the adjective depending on the question type—which was the decisive criterion, irrespective of any additional information, even if it did not match the sentence content. For example, if the question targeted the location *into the wardrobe*, as in (b), answers such as *wardrobe*, *closet*, *wadorbe*, *big wardrobe*, *blue wardrobe*, *Anna put the green skirt into a wardrobe* would all be coded as correct. In contrast, answers such as *on the bed*, *Anna put the skirt into*, and *beige something* would be coded as incorrect.

All participants were presented with the same sentences. Question types were assigned in a Latin-squared design and sentence order and the order of questions was fully randomized for each participant.

We performed two blocks of experiments (see Table 2 for overview). The first block of three experiments was based on our preregistered predictions, using sentences as demonstrated above. In the first block of experiments, we wanted to see to what degree the focus of the questions (noun vs. adjective) affects the adaptation / learning of the participants. To this end, we manipulated the distribution of noun vs. adjective questions in the experiments. In experiment 1, all questions were equally distributed. In experiment 2, questions targeting the adjective occurred in 25% of the cases. In experiment 3, questions targeting the adjective occurred in 75% of the cases.

**Table 2.** Overview of the six experiments conducted in this study. The ratio of question types refers to the number of adjective-targeting and noun-targeting questions.

Experiment	*N* participants	Sentence length	Ratio of question types
Experiment 1	230	short	48:48 (50:50%)
Experiment 2	223	short	72:24 (75:25%)
Experiment 3	207	short	24:72 (25:75%)
Experiment 4	237	long	48:48 (50:50%)
Experiment 5	329	long	72:24 (75:25%)
Experiment 6	292	long	24:72 (25:75%)

A preliminary analysis of these three experiments indicated that participants performed almost at ceiling in their response accuracies, especially for nouns. This is why we devised a second block of three experiments with the same distribution of questions (same, 25% adjectives, 75% adjectives). In this second block, we provided longer and more complex sentences that were constructed by taking the sentences from experiments 1 to 3 and adding additional material. The main clause remained almost the same, except for adding an adverb of manner (e.g., *rychle*, ‘quickly’) immediately before the verb. To the end of each sentence, an additional temporal clause was then added. For example, the sentence *Anna nacpala obstarožní sukni do béžové skříně.* (‘Anna stuffed the outmoded skirt into the beige wardrobe.’) was transformed into *Anna rychle nacpala obstarožní sukni do béžové skříně, když na jaře procházela šatník* (‘Anna quickly stuffed an outmoded skirt into a beige wardrobe when she was going through her closet in the spring.’). By making the sentences longer, we expected participants’ response accuracy to drop, which would in turn allow us to observe adaptation effects across the experiments without the presence of a ceiling effect.

### Statistical Approach and Variables

We used the lme4 package (version 1.1-33; Bates et al., 2014) in the programming language R (version 3.9.0) for statistical analysis. We used linear mixed-effects regression when investigating reaction times and generalized linear mixed-effects regression when investigating accuracies.

In the analyses for both reaction times (RTs) and recall accuracy, we first conducted a general model comparing the results of the six experiments. Since there was a clear difference between the first three experiments (which employed shorter sentences) and the last three experiments (which used longer sentences) in terms of both RTs and recall accuracy, we then conducted analyses for these two blocks of experiments separately.

Altogether we tested the following set of variables as predictors:

Sentence length is one of the two variables distinguishing between the six experiments. It has two values: short (Experiments 1, 2, and 3) vs. long (Experiments 4, 5, and 6). In the analyses, it was sum-contrast coded with long sentences coded as 0.5 and short coded as −0.5.

The second variable distinguishing between the six experiments was the Ratio of question types. It has three values: 25% adjective-targeting questions, 50% adjective-targeting questions and 75% adjective-targeting questions. It was coded using repeated contrasts, resulting in two comparisons: (i) 25% adjective-targeting questions vs. 50% adjective-targeting questions and (ii) 50% adjective-targeting questions vs. 75% adjective-targeting questions.

The crucial predictor involved in both the analysis of RTs and in the analysis of recall accuracy was Trial. This represents the trial number across the experiment. Trial was centered and scaled for the analysis.

In the analysis of reaction times, we further examined the effects of Information type, which specified two types of target words: adjectives and nouns. This variable was sum-contrast coded with adjectives coded as 0.5 and nouns coded as −0.5. The last factor used in the reaction time analysis was Word type, which captured the distinction between words targeted by the comprehension questions and other words in the sentence that were never targeted by a comprehension question. Since longer sentences contained more words than the shorter sentences, we used only those context words that were presented in all six experiments. This factor was also sum-contrast coded (with target words coded as 0.5, and context words as −0.5).

The analysis of recall accuracy used two additional variables. Question represents what the target of the question was: noun (either the object noun, or locative noun) or adjective (either modifying the object, or modifying the locative noun). Based on our hypothesis that there would be an inherent decline between the target types, this predictor was sum-contrast coded with questions targeting adjectives coded as 0.5 and question targeting nouns as −0.5.

Focus specifies whether in the presented sentence, the question target is focused or not. It was sum-contrast coded with focused value coded as 0.5 and not focused as −0.5.

Regarding our dependent variables, we investigated how the predictors discussed above were related to response accuracies and reaction times. For the analysis, we trimmed the reaction times based on these three steps. First, we excluded all RTs below 100 ms. Second, we log-transformed RTs and calculated the mean and its standard deviation. As the higher cut-off point, we then used 2.5 standard deviations for the mean. This was done for each experiment separately. We will discuss the specifics of our dependent variables in the Results section.

## RESULTS

### Reaction Times

**Figure 1.**
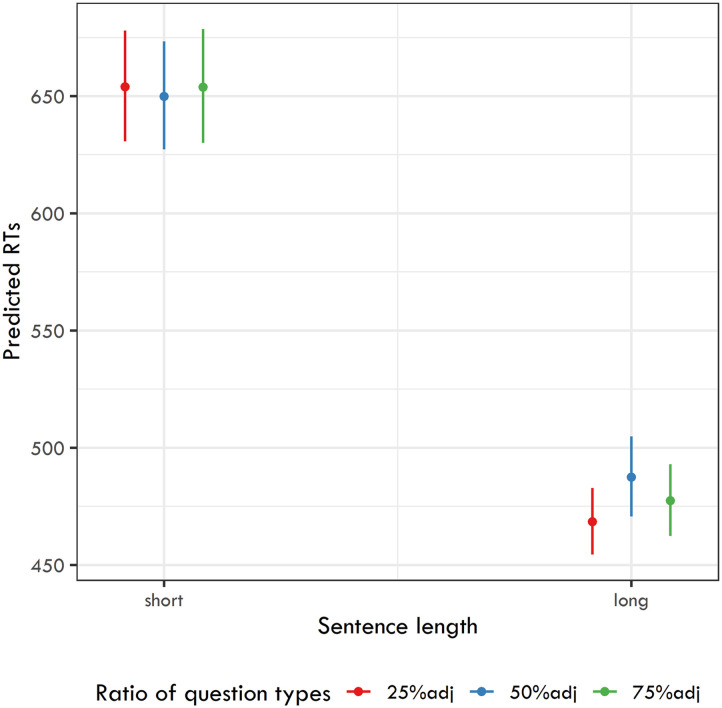
Back-transformed predicted reaction times for target words in the six experiments.

In the initial analysis, we used a linear mixed-effects model (Bates et al., 2014) to examine the differences among the six experiments. The model included participant and item as random effects (without a random slope) and Sentence length and Ratio of question types as fixed effects in interaction. As the dependent variable, we used log-transformed reaction times for the four types of information targeted by the comprehension questions (i.e., direct object nouns, adjectives modifying object nouns, nouns in the locative prepositional phrases, and their modifying adjectives). The model yielded a significant effect of Sentence length (*β* = −0.312, *SE* = 0.016, *t* = −19.7, *p* < 0.001), indicating that reaction times (RTs) on target words in experiments using longer sentences were significantly faster. No other effect has reached significance. The predicted back-transformed RTs are shown in Figure 1.

Since we established a robust difference between the experiments based on the length of the stimuli used, we then analyzed these two groups of experiments separately.

In the subsequent analysis, we focused on the words targeted by the comprehension questions, examining to what extent their RTs were influenced by (i) Ratio of question types, (ii) Information type, and (iii) Trial. The linear mixed-effects model included the fixed effects of the Information type in interaction with Trial, with these two effects nested under the Ratio of question types. The random effects were participant and item (without random slopes due to convergence problems).

**Figure 2.**
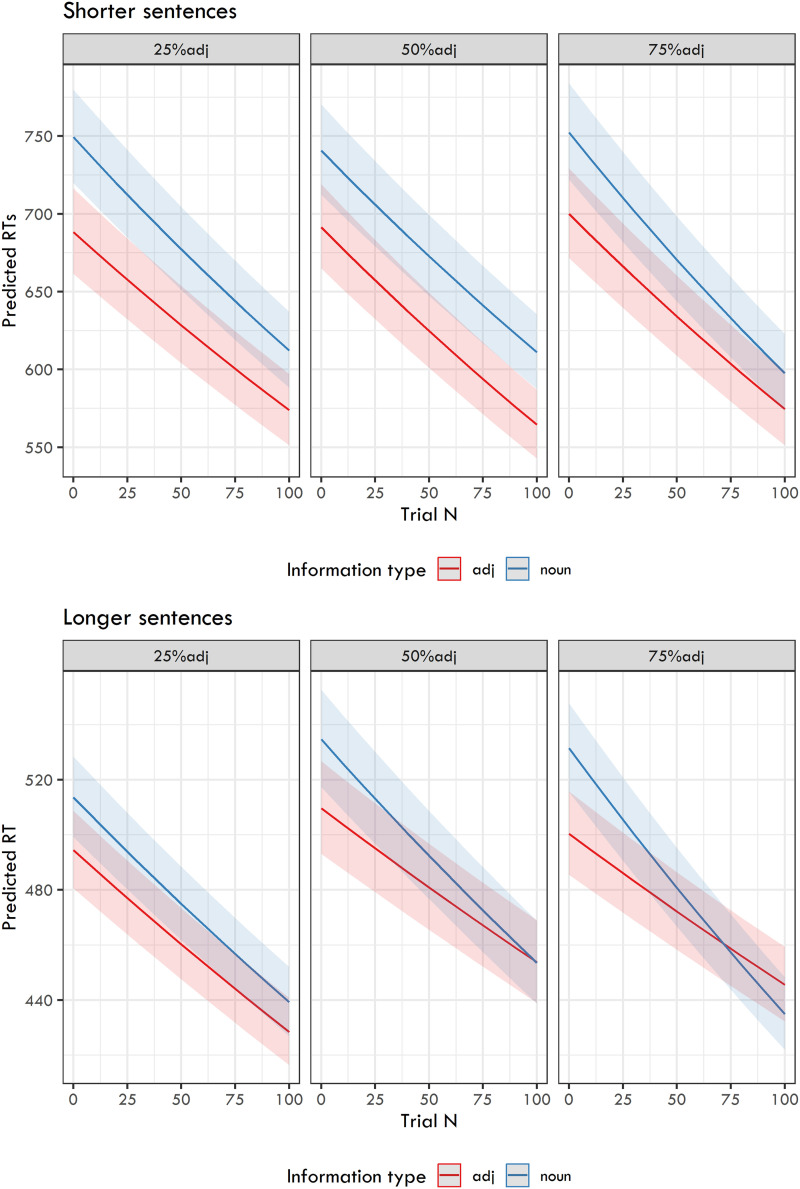
Back-transformed predicted reaction times from the linear mixed-effects models capturing the relation between the Ratio of question types, Trial, and Information type.

The model results (estimates for fixed effects, their confidence intervals, *t*-values and *p*-values) are presented in Table 3 and the back-transformed predicted RTs are shown in Figure 2.

**Table 3.** Results for fixed effects from the linear mixed effect models fitting reaction times on target words. Ratio = Ratio of question types, InfType = Information type.

Predictor	**Short sentences**			**Long sentences**		
	Estimate [95% CI]	*t*-value	*p*-value	Estimate [95% CI]	*t*-value	*p*-value
Intercept	6.481 [6.457; 6.506]	509.756	<0.001	6.169 [6.150; 6.189]	623.7	<0.001
Ratio 50vs25	−0.006 [−0.058; 0.046]	−0.234	0.815	0.04 [−0.000; 0.080]	1.951	0.051
Ratio 75vs50	0.006 [−0.047; 0.059]	0.22	0.826	−0.021 [−0.062; 0.020]	−0.992	0.321
Ratio [25%adj] / InfType	−0.075 [−0.080; −0.070]	−28.878	<0.001	−0.032 [−0.036; −0.027]	−14.619	<0.001
Ratio [50%adj] / InfType	−0.074 [−0.079; −0.069]	−28.973	<0.001	−0.024 [−0.029; −0.019]	−9.535	<0.001
Ratio [75%adj] / InfType	−0.056 [−0.062; −0.051]	−20.98	<0.001	−0.019 [−0.024; −0.015]	−8.317	<0.001
Ratio [25%adj] / Trial	−0.053 [−0.056; −0.051]	−40.85	<0.001	−0.041 [−0.044; −0.039]	−38.357	<0.001
Ratio [50%adj] / Trial	−0.055 [−0.057; −0.052]	−42.914	<0.001	−0.039 [−0.041; −0.036]	−30.441	<0.001
Ratio [75%adj] / Trial	−0.059 [−0.062; −0.057]	−44.074	<0.001	−0.044 [−0.046; −0.042]	−37.761	<0.001
Ratio [25%adj] / InfType * Trial	0.006 [0.000; 0.011]	2.142	0.032	0.004 [−0.001; 0.008]	1.671	0.095
Ratio [50%adj] / InfType * Trial	−0.003 [−0.008; 0.002]	−1.131	0.258	0.013 [0.008; 0.018]	5.254	<0.001
Ratio [75%adj] / InfType * Trial	0.009 [0.004; 0.014]	3.376	0.001	0.023 [0.019; 0.028]	10.077	<0.001

There are several important findings. First, the Trial effects are very robust in both blocks of experiments. This means that participants’ reactions became gradually faster over the course of the experiments. Second, information type was a highly significant predictor in all experiments, with RTs for adjectives generally faster than those for nouns. The last and rather interesting finding is the interaction between information type and trial number. This interaction is highly significant when 75% adjective-targeting questions were used (in both short and long sentence experiments) and when 50% adjective-targeting questions were used (in the long sentence experiment). This means that the gradual speed-up for adjectives was lower than for nouns. In other words, in experiments where participants were more frequently asked about adjectival information, the RTs for adjectives did not decrease as much as the RTs for nouns. This is especially evident in Figure 2, particularly for the longer sentence experiments.

**Figure 3.**
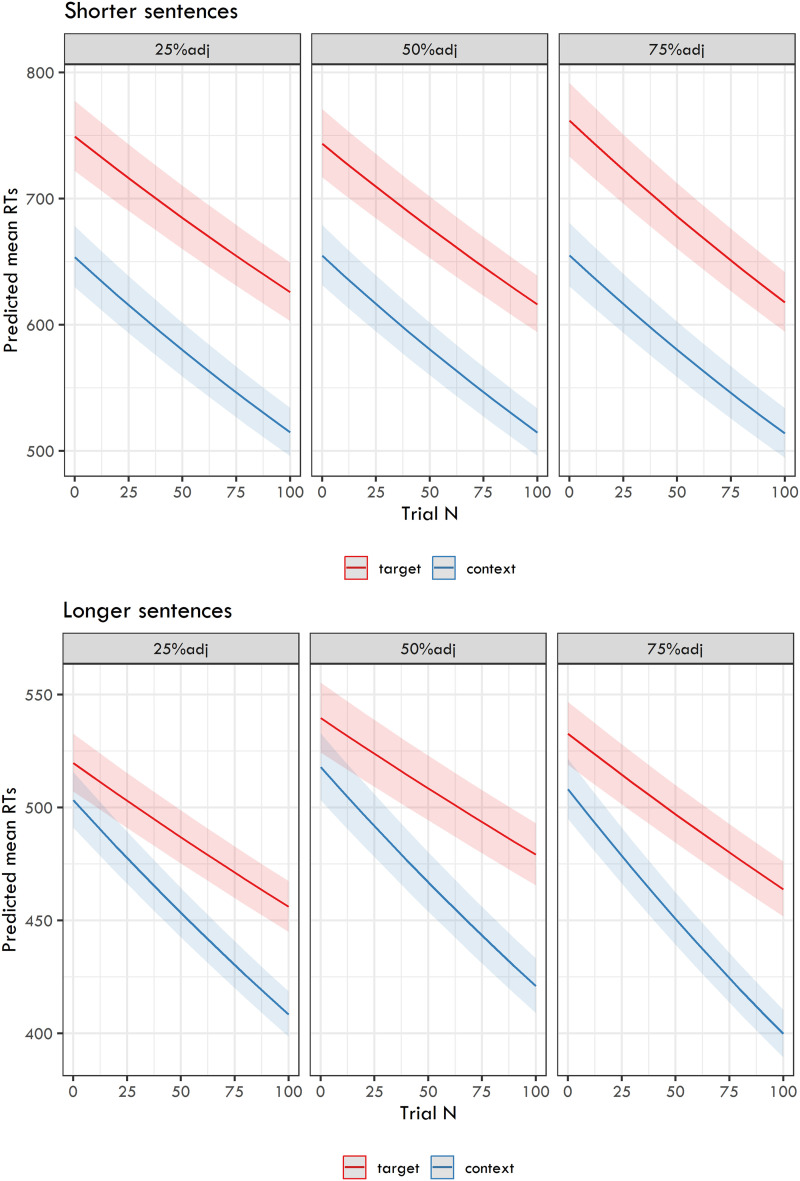
Back-transformed predicted mean RTs from the linear mixed-effects models capturing the relation between the Ratio of question types, Trial, and Word Type.

The final analysis focused on the difference in RTs between the words targeted by comprehension questions and other words in the sentence. As the dependent variable, we used the log-transformed mean RTs for target words and for context words calculated per participant, item, and trial number. The fixed effects included Trial, Ratio of question types, and Word type. The model results (estimates for fixed effects, their confidence intervals, *t*-values, and *p*-values) are presented in Table 4, and the back-transformed predicted RTs are shown in Figure 3.

**Table 4.** Results for fixed effects from the linear mixed effect models fitting mean reaction times on target vs. context words. Ratio = Ratio of question types, WordType = Word Type (context vs. target words).

Predictor	**Short sentences**			**Long sentences**		
	Estimate [95% CI]	*t*-value	*p*-value	Estimate [95% CI]	*t*-value	*p*-value
Intercept	6.447 [6.425; 6.470]	564.532	<0.001	6.169 [6.153; 6.185]	750.621	<0.001
Ratio 50vs25	−0.005 [−0.055; 0.044]	−0.212	0.832	0.036 [0.001; 0.072]	2.015	0.044
Ratio 75vs50	0.007 [−0.044; 0.057]	0.256	0.798	−0.029 [−0.065; 0.008]	−1.548	0.122
Ratio [25%adj] / WordType	0.165 [0.161; 0.170]	69.844	<0.001	0.07 [0.067; 0.074]	35.656	<0.001
Ratio [50%adj] / WordType	0.153 [0.148; 0.157]	65.772	<0.001	0.084 [0.080; 0.089]	36.161	<0.001
Ratio [75%adj] / WordType	0.167 [0.162; 0.172]	68.482	<0.001	0.097 [0.093; 0.101]	45.77	<0.001
Ratio [25%adj] / Trial	−0.058 [−0.060; −0.056]	−48.956	<0.001	−0.047 [−0.049; −0.045]	−47.774	<0.001
Ratio [50%adj] / Trial	−0.059 [−0.062; −0.057]	−51.104	<0.001	−0.045 [−0.048; −0.043]	−38.906	<0.001
Ratio [75%adj] / Trial	−0.063 [−0.065; −0.060]	−51.377	<0.001	−0.053 [−0.055; −0.050]	−49.664	<0.001
Ratio [25%adj] / WordType * Trial	0.016 [0.012; 0.021]	6.899	<0.001	0.022 [0.018; 0.026]	11.023	<0.001
Ratio [50%adj] / WordType * Trial	0.015 [0.010; 0.019]	6.328	<0.001	0.025 [0.020; 0.029]	10.558	<0.001
Ratio [75%adj] / WordType * Trial	0.009 [0.004; 0.014]	3.756	<0.001	0.028 [0.024; 0.032]	13.32	<0.001

The models again document a robust effect of Trial in all six experiments. Moreover, there is a robust difference in RTs between the two word types—context words have faster RTs than target words. Crucially, the models yield an interaction between Trial and Word Type in all six experiments. This indicates that the RTs for context words tend to speed up faster during the experiment than the RTs for target words.

### Recall Accuracy

Analogous to the analysis of RTs, we first ran a model targeting the differences between the experiments. We ran a logit mixed-effects model with participant and item as random effects (without a random slope) and Sentence length and Ratio of question types as fixed effects in interaction.

**Figure 4.**
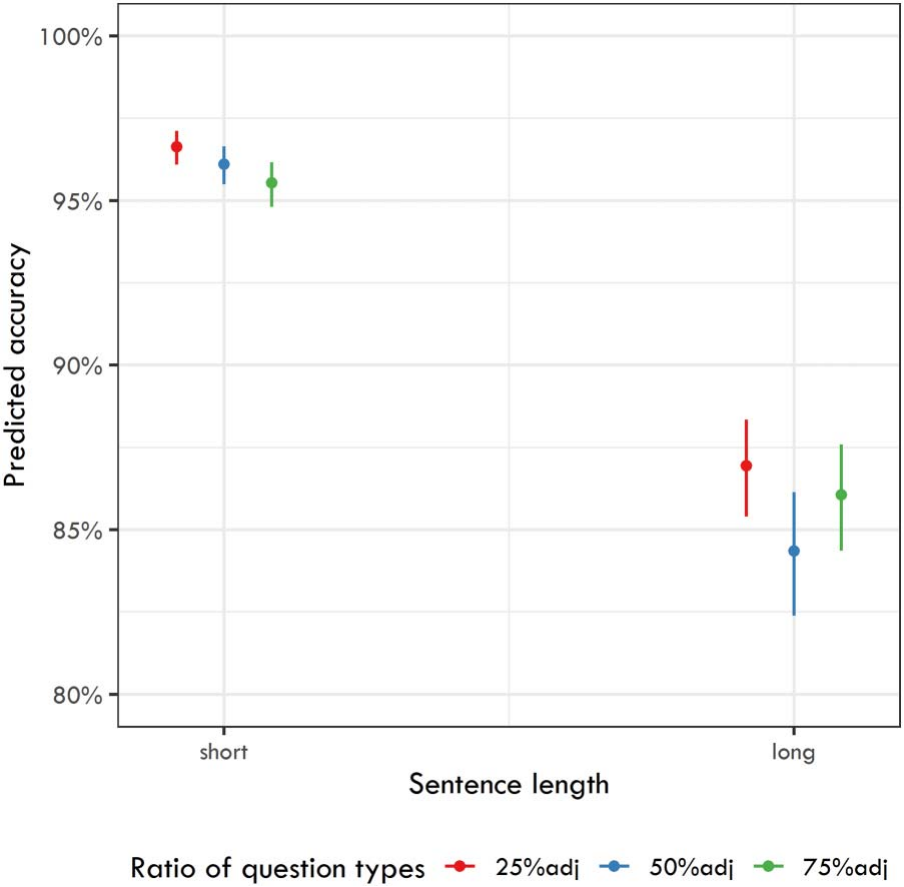
Predicted recall accuracies for the six experiments.

The dependent variable was answer correctness (with two values: correct vs. incorrect). The model yielded a significant effect of Sentence length (*β* = −1.409, *SE* = 0.08, *z* = −17.598, *p* < 0.001), indicating that experiments using longer sentences have generally lower recall accuracy. Additionally, the model yielded two more significant results. First, experiments with 50% adjective-targeting questions had generally worse recall accuracy than those with 25% adjective-targeting questions (*β* = −0.181, *SE* = 0.059, *z* = −3.055, *p* = 0.002). Second, there was a significant interaction between Sentence length and the second comparison of Ratio of question types (*β* = 0.279, *SE* = 0.12, *z* = 2.321, *p* = 0.02), indicating that recall accuracy in the experiment using 75% adjective-targeting questions was better with longer sentences but worse with shorter sentences (see Figure 4 for the predicted accuracies of the six experiments).

As a second step, we fitted separate models for the two blocks of experiments (Experiments 1, 2, and 3, which used shorter sentences, and Experiments 4, 5, and 6, which employed longer sentences). The dependent variable was response correctness. The fixed effects included Trial, Focus, and Question in interaction, and these effects were nested under the Ratio of question types. The results for fixed effects are presented in Table 5, and the predicted accuracies are shown in Figure 6.

**Table 5.** Results for fixed effects from the generalized linear mixed effect models fitting recall accuracy as a function of Question, Focus, and Trial.

Predictor	**Short sentences**			**Long sentences**		
	Odds Ratio [95% CI]	*z*-value	*p*-value	Odds Ratio [95% CI]	*z*-value	*p*-value
Intercept	27.578 [24.096, 31.564]	48.161	<0.001	5.689 [5.146, 6.290]	33.962	<0.001
Ratio 50vs25	1.089 [0.908, 1.307]	0.923	0.356	1.063 [1.007, 1.122]	2.196	0.028
Ratio 75vs50	0.864 [0.718, 1.040]	−1.544	0.123	1.281 [1.207, 1.360]	8.136	<0.001
Ratio [25%adj] / Focus	2.583 [2.240, 2.979]	13.034	<0.001	1.453 [1.358, 1.555]	10.82	<0.001
Ratio [50%adj] / Focus	2.4 [2.096, 2.748]	12.685	<0.001	1.397 [1.296, 1.506]	8.71	<0.001
Ratio [75%adj] / Focus	2.583 [2.220, 3.005]	12.29	<0.001	1.258 [1.155, 1.370]	5.268	<0.001
Ratio [25%adj] / Question	0.362 [0.313, 0.417]	−13.966	<0.001	0.297 [0.277, 0.318]	−35.044	<0.001
Ratio [50%adj] / Question	0.463 [0.404, 0.530]	−11.143	<0.001	0.396 [0.368, 0.428]	−24.037	<0.001
Ratio [75%adj] / Question	0.899 [0.773, 1.046]	−1.381	0.167	0.592 [0.544, 0.645]	−12.017	<0.001
Ratio [25%adj] / Trial	1.143 [1.065, 1.227]	3.708	<0.001	1.469 [1.420, 1.520]	22.138	<0.001
Ratio [50%adj] / Trial	1.178 [1.101, 1.260]	4.729	<0.001	1.527 [1.471, 1.586]	22.14	<0.001
Ratio [75%adj] / Trial	1.115 [1.034, 1.202]	2.832	0.005	1.566 [1.501, 1.634]	20.579	<0.001
Ratio [25%adj] / Focus * Question	1.482 [1.114, 1.971]	2.701	0.007	0.947 [0.827, 1.084]	−0.786	0.432
Ratio [50%adj] / Focus * Question	1.687 [1.287, 2.210]	3.791	<0.001	1.158 [0.996, 1.346]	1.911	0.056
Ratio [75%adj] / Focus * Question	2.009 [1.485, 2.719]	4.522	<0.001	1.137 [0.958, 1.349]	1.472	0.141
Ratio [25%adj] / Focus * Trial	1.124 [0.975, 1.296]	1.614	0.107	1.035 [0.967, 1.108]	0.993	0.321
Ratio [50%adj] / Focus * Trial	0.933 [0.814, 1.069]	−1.004	0.315	0.98 [0.910, 1.057]	−0.515	0.606
Ratio [75%adj] / Focus * Trial	1.138 [0.979, 1.323]	1.683	0.092	0.997 [0.915, 1.086]	−0.074	0.941
Ratio [25%adj] / Question * Trial	1.469 [1.275, 1.693]	5.311	<0.001	1.113 [1.040, 1.192]	3.086	0.002
Ratio [50%adj] / Question * Trial	1.116 [0.974, 1.278]	1.576	0.115	1.116 [1.035, 1.203]	2.867	0.004
Ratio [75%adj] / Question * Trial	1.132 [0.974, 1.316]	1.612	0.107	1.195 [1.097, 1.301]	4.078	<0.001
Ratio [25%adj] / Focus * Question * Trial	1.308 [0.985, 1.738]	1.854	0.064	1.034 [0.902, 1.185]	0.483	0.629
Ratio [50%adj] / Focus * Question * Trial	1.135 [0.865, 1.490]	0.913	0.361	1.011 [0.870, 1.174]	0.14	0.889
Ratio [75%adj] / Focus * Question * Trial	1.08 [0.799, 1.459]	0.501	0.616	1.105 [0.931, 1.310]	1.144	0.253

The models yielded several important findings. First, Trial was significant in all six experiments, indicating that response accuracy gradually increased over the course of an experiment. Second, the effect of Focus was also significant in all six experiments, meaning that focused information had higher recall accuracy than non-focused information. Third, Question was significant in all but one experiment (i.e., Experiment 3, which employed 75% adjective-targeting questions with shorter sentences). This indicates that adjective-targeting questions have lower recall accuracy than noun-targeting questions. Fourth, the interaction between Question and Focus was significant in all experiments using shorter sentences, but not in any experiments using longer sentences. In other words, focused words seem to have a more prominent position in shorter sentences than in the longer ones and the difference in recall between focused and non-focused positions is thus greater for shorter than for longer sentences. Fifth, the interaction between Question and Trial was significant in all three experiments using longer sentences and in the experiment using shorter sentences with 25% adjective-targeting questions. Other effects did not reach significance. Moreover, there was a significant effect of the second comparison of the Ratio of question types for longer sentences, which means that experiment using 75% adjective-targeting questions had better recall accuracy than the experiment using 50% adjective-targeting questions.

**Figure 5.**
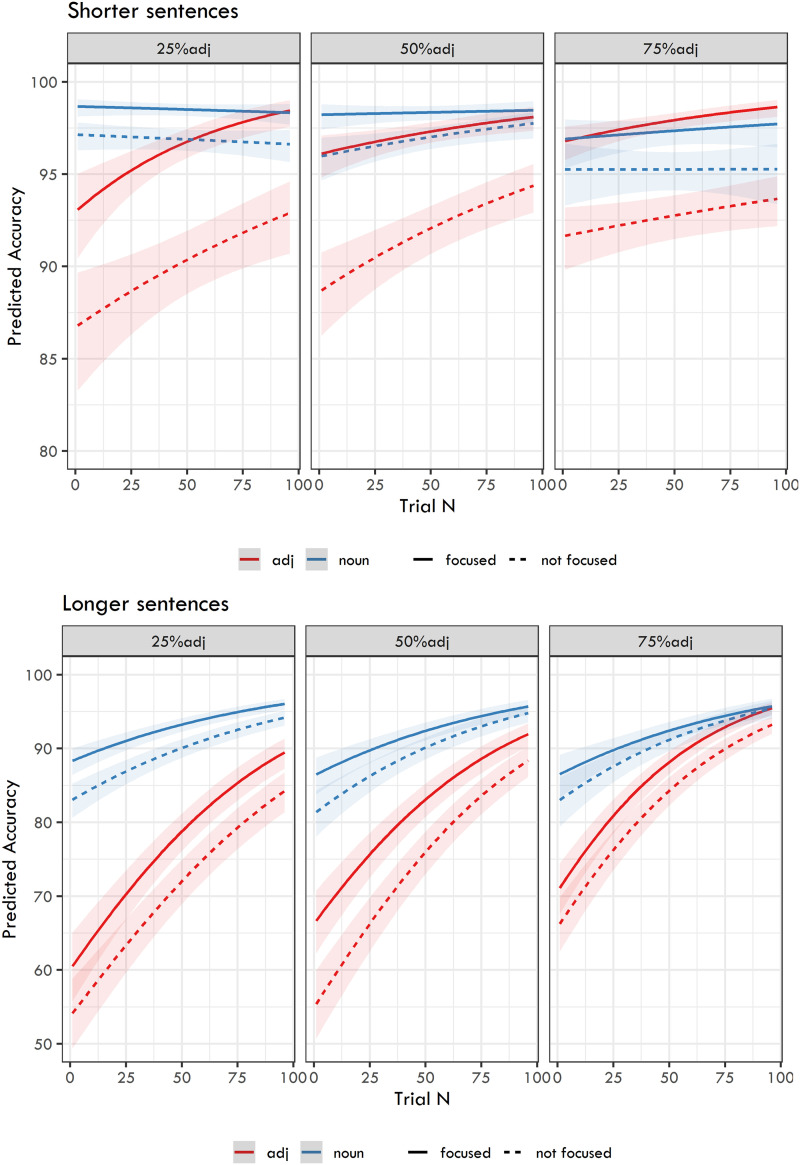
Predicted recall accuracy from the logit mixed-effects models capturing the relation between the Ratio of question types, Trial, Question, and Focus.

The final analysis considered the relationship between recall accuracy and reaction times. In the model, we used scaled and centered RTs for the target word in interaction with Trial, and we nested this interaction under Question. Again, we modeled the results for shorter and longer sentences separately. Table 6 captures the fixed effects of these models, and Figure 5 presents the predicted accuracies.

**Table 6.** Results for fixed effects from the generalized linear mixed effect models fitting recall accuracy as a function of Question, RT, and Trial.

Predictor	**Short sentences**			**Long sentences**		
	Odds Ratio [95% CI]	*z*-value	*p*-value	Odds Ratio [95% CI]	*z*-value	*p*-value
Intercept	27.332 [23.936; 31.210]	48.867	<0.001	7.115 [6.401; 7.908]	36.386	<0.001
Question	0.470 [0.434; 0.510]	−18.329	<0.001	0.371 [0.353; 0.388]	−41.077	<0.001
Question [adj] × RT	1.256 [1.178; 1.340]	6.973	<0.001	1.144 [1.104; 1.185]	7.457	<0.001
Question [noun] × RT	1.189 [1.102; 1.283]	4.461	<0.001	1.123 [1.075; 1.172]	5.262	<0.001
Question [adj] × Trial	1.242 [1.186; 1.301]	9.183	<0.001	1.751 [1.703; 1.801]	39.223	<0.001
Question [noun] × Trial	1.028 [0.968; 1.091]	0.892	0.372	1.458 [1.411; 1.507]	22.338	<0.001
Question [adj] × RT × Trial	1.052 [1.005; 1.102]	2.185	0.029	1.084 [1.054; 1.115]	5.594	<0.001
Question [noun] × RT × Trial	0.988 [0.931; 1.049]	−0.387	0.699	1.037 [1.003; 1.073]	2.113	0.035

As expected, the models yielded a highly significant effect of Question, i.e., higher recall accuracy for nouns. Importantly, both adjective and noun recall were influenced by RTs in both groups of experiments. The models show that the longer the RTs on the word targeted by the question, the better the recall accuracy. Trial was also highly significant, with the exception of noun recall for shorter sentences. This can be interpreted as a ceiling effect: recall of nouns in shorter sentences was already so high at the beginning of the experiments that it could not improve much during the experiments (see also Figure 5 for a visualization). The last documented significant effect was an interaction between RT and Trial for adjective recall in both shorter and longer sentences, and a mildly significant effect of this interaction for nouns in longer sentences, meaning that the effects of RTs on recall accuracy grew even stronger during the course of the experiment.

**Figure 6.**
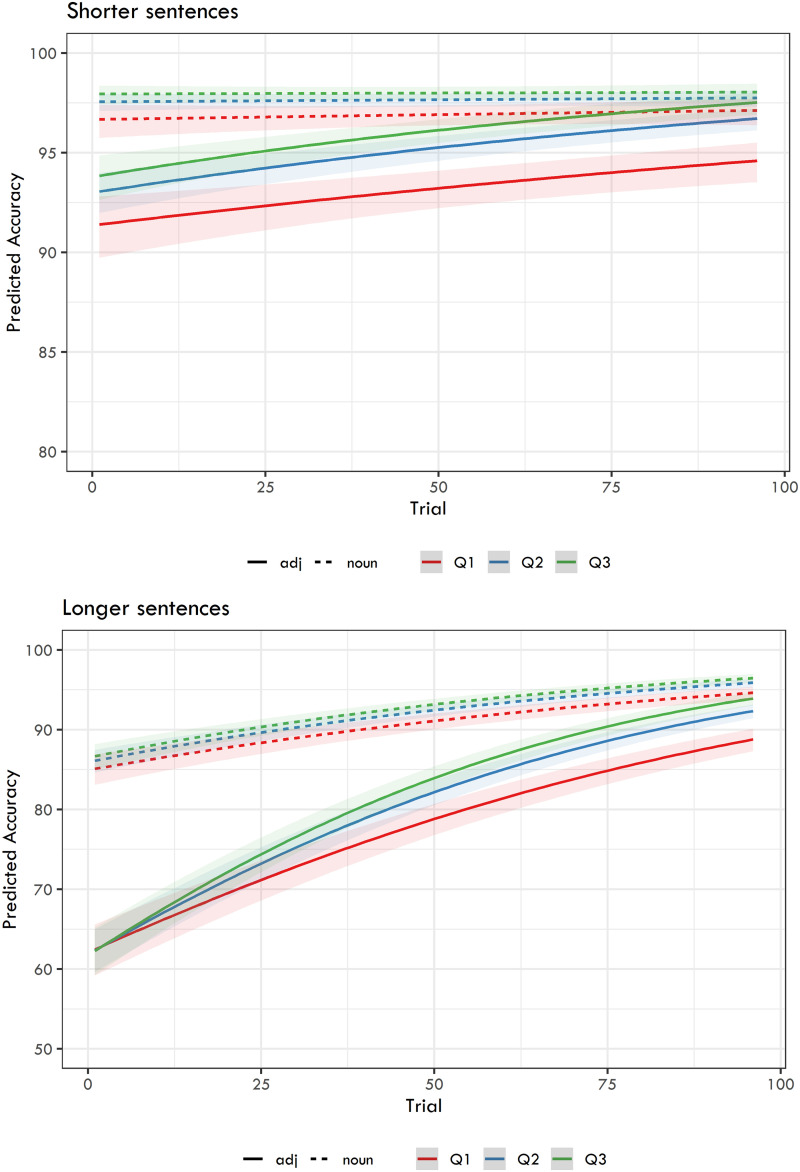
Predicted recall accuracy from the logit mixed-effects models capturing the relation between the Question, RTs, and Trial. Q1 = 1st quartile of RTs, Q2 = median RT, Q3 = 3rd quartile of RTs.

## EFFECT SUMMARY

Since the analyses of RTs and recall accuracy were rather complex, we present a brief summary of documented effects in Table 7.

**Table 7.** Summary of significant effects from the models of RTs and recall accuracy. NA = not analyzed.

Predictor	Values	RTs	Recall
Sentence length	long vs. short	highly significant	highly significant
Ratio of questions	25% adj vs. 50% adj vs. 75% adj	no effect	difference for 50% adj and 75%, in long sentences
Trial	numeric	highly significant	highly significant
Information type	adjective vs. noun	highly significant	NA
Information type * Trial	adjective * trial	highly significant for 75% adj, in long sentences also for 50% adj	NA
Word type	target vs. context	highly significant	NA
Word type * Trial	target * trial	highly significant	NA
Focus	focused vs. not focused	NA	highly significant
Question	adjective vs. noun	NA	highly significant (with an exception in short sentences, 75% adj)
Focus * Question	focused * adjective	NA	significant for short sentences
Question * Trial	adjective * trial	NA	significant for long sentences, and for short sentences, 25% adj
RT	numeric	NA	highly significant
RT * Trial	numeric * numeric	NA	significant with and exception of nouns in shorter sentences

## DISCUSSION

In this paper, we examined the learning-based (Heitmeier et al., 2023; Hodapp & Rabovsky, 2024; Tomaschek et al., 2024) and motivation-based explanations (cf. Christianson et al., 2022) of a well-documented phenomenon of progressive acceleration in processing speed throughout an experimental task, commonly known as task adaptation. While the learning-based view predicts both progressively faster processing and higher recall accuracy, the motivation-based view expects that while processing gets faster, recall accuracy gradually drops. To test these assumptions, we undertook six self-paced reading experiments, varying the structure of comprehension questions and stimuli length. Crucially, our findings document substantial learning effects over the course of these experiments, with participants’ processing getting gradually faster and more precise.

In testing the learning effects, we examined immediate recall of information conveyed by adjectives in attributive position, locative nouns and nouns which had a role of direct objects. Consistent with the findings of Chromý and Vojvodić (2024), we documented significant differences in recall of information conveyed by adjectives and nouns in all six experiments. Information conveyed through attributive adjectives consistently exhibited a lower recall rate compared to information conveyed by nouns.

Importantly, Chromý and Vojvodić (2024) proposed that such systematic differences in recall could be attributed to selective attention mechanisms in reading. However, they could not directly test this claim as it primarily focused on offline recall and did not capture reaction times for specific sentence regions. In contrast, our present study employed word-by-word self-paced reading, enabling us to correlate reaction times with response accuracy. Interestingly, we discovered a positive relationship between longer reaction times in target regions and improved recall accuracy. This provides direct evidence supporting an attention-based explanation for recall differences.

The dynamics of attention appear to be influenced by yet another type of cue. Participants seem to learn what kind of information is targeted by the comprehension questions and adapt their reading behavior accordingly. In the analysis of reaction times, we observed that the general tendency to steadily accelerate reading throughout the course of an experiment is more pronounced for those parts of the sentences that are unrelated to the comprehension questions than for those specifically targeted. Moreover, the gradual acceleration observed during the experiment is also affected by the ratio of question types. It appears that participants who received a higher proportion of adjective-targeting questions exhibited a smaller reduction in their response times for adjectives compared to nouns.

Additionally, the recall accuracy analysis revealed intriguing differences in recall attributable to the distribution of comprehension question types. The more adjective-targeting questions participants received, the better their recall accuracy for this type of information. Evidently, participants exhibit a strategic allocation of attention to segments of the sentence they learn to recognize as crucial for accomplishing their task successfully.

In addition to uncovering differences in recall based on the type of information, our study also documented a distinct relationship with the information structure of the sentence. When the information was focused within the sentence, it exhibited a higher recall rate. This observation aligns well with prior research on the role of focus (Birch & Garnsey, 1995; Birch & Rayner, 1997; Lowder & Gordon, 2015). Readers thus seem to employ focus as a cue for determining the importance of information which is then translated to their ability to recall this information.

It could be argued that our results cannot be generalized due to the specific methodology employed in this paper. Indeed, we deliberately deviated from standard practices in sentence processing research. Unlike most studies, we did not use filler items and opted for open-ended questions instead of the commonly used yes-no questions.

We omitted filler items to control for learning effects throughout the experiment. Including filler items of various syntactic structures, lengths, and types of comprehension questions would have introduced an uncontrolled confound. To avoid this and maintain a simple design, we excluded them. Consequently, our results can be seen as a product of “learning without obstacles”, and the learning effects observed here may be stronger than those found in studies with more complex stimuli designs. Learning is defined as “a process that reapportions attentional/representational resources in order to maximize future predictive success” (Ramscar & Baayen, 2013). Each filler item in the experiment would update the participant’s predictions, potentially altering their expectations for subsequent items and even causing unlearning in some cases (Vujović et al., 2021). Therefore, it would be interesting to systematically examine learning effects in the presence of intervening filler materials. Crucially, we believe it is methodologically undesirable to test learning effects without accounting for the presence and structure of fillers, as has been typically done in studies on syntactic adaptation (e.g., Dempsey et al., 2024; Fine et al., 2013).

The use of open-ended questions raises another methodological consideration. We chose them to examine what participants actually recall from the sentences they just read, an interesting problem in itself (Chromý & Vojvodić, 2024). Open-ended questions also address the main issue with yes-no questions: the high probability of guessing the correct answer. With only two possible answers, the chance of guessing correctly is 50%, and this probability increases if the participant processes even a small amount of information from the sentence. An inattentive reader may still achieve high response accuracy for unambiguous and plausible sentences with yes-no questions. In contrast, open-ended questions require participants to actively formulate their answers without predefined options, which is more challenging. The impact of using these two types of questions was clearly demonstrated by Ceháková and Chromý (2023), who found significantly lower response accuracy in experiments when participants had to answer open-ended questions rather than respond yes-no. We thus believe that open-ended questions provide a better understanding of the “depth of comprehension” (Ferreira & Yang, 2019) and offer more insight into the learning effects throughout an experiment than traditional yes-no questions.

One of the reviewers suggested using a drift diffusion model (DDM, cf. Ratcliff & McKoon, 2008) to analyze RTs and accuracies simultaneously. Indeed, the DDM offers a comprehensive framework that integrates both measures, potentially providing deeper insights into the cognitive processes underlying task performance, including the separation of non-decision time from decision-related processes. Such an approach could illuminate whether observed speedups are attributable to changes in non-decision time, higher cognitive processes, or a combination of both. However, while this approach could provide valuable insights into the underlying cognitive processes, implementing and validating the model requires substantial additional resources. Moreover, the application of DDMs is not without criticisms (Jones & Dzhafarov, 2014). Therefore, while we acknowledge the possible benefits of using a DDM, we believe that our current methodological approach provides a valid and interpretable analysis of the data. We encourage future research to explore this modeling technique to further elucidate the mechanisms at play. For the present study, we have chosen to focus on more traditional methods that allow for a clear and straightforward interpretation of the results.

The present findings have important implications for the general methodology of reading research, for sentence processing studies, and in particular, for dealing with conflicting results of different studies. First, it is clear that the length of an experiment (number of trials used) can significantly affect its results. This point has often been made, and is often highlighted in single-trial experiments (Laurinavichyute & von der Malsburg, 2022, 2024). But what we suggest here is crucial: these effects must be accounted for in comparisons between results of different experiments. Which is to say that what is important is not only how many experimental sentences are used, but also the number of fillers. Second, we offer robust evidence that the distribution of comprehension question targets used in an experiment will influence the actual reading behavior of participants. Accordingly, researchers should think carefully about the kinds of comprehension questions they ask, both in terms of the experimental items and fillers, and the structure of the set of comprehension questions, and that the details of both should be reported in published studies.

The third methodological consequence is related to a rather accidental finding of the current research. Our data reveal a strong correlation between sentence length and the reaction time for each word, with longer sentences exhibiting shorter reaction times. This finding bears a resemblance to the well-established Menzerath’s Law (Altmann, 1980; Menzerath, 1954; Milička, 2014) which states that the longer words or phrases are, the smaller are the segments they are made of. We posit that readers may strategically allocate their cognitive resources during reading, particularly when they have information about sentence length, as is the case in moving window version of the self-paced reading task. However, it is imperative to subject this finding to more comprehensive scrutiny, ideally exploring intraindividual variability by incorporating both short and long sentences within a single experiment. Furthermore, it is crucial to test this phenomenon within a more natural reading paradigm, such as eye-tracking. Crucially, the possible presence of this effect suggests that the overall length of a sentence can influence the reading times of any smaller regions under examination.

## ACKNOWLEDGMENTS

We would like to thank all of our participants from Charles University and the LABELS Lab whose participant pool we were able to use. Next, our gratitude goes to the two anonymous reviewers of this article who helped improve it.

## FUNDING INFORMATION

This work was supported by the European Regional Development Fund project “Beyond Security: Role of Conflict in Resilience-Building” (reg. no.: CZ.02.01.01/00/22_008/0004595). The main author was also supported by the Charles University institutional program Cooperatio.

## AUTHOR CONTRIBUTIONS

Jan Chromý: Conceptualization; Data curation; Formal analysis; Funding acquisition; Investigation; Methodology; Resources; Software; Visualization; Writing – Original draft; Writing – Review & editing. Fabian Tomaschek: Conceptualization; Data curation; Formal analysis; Methodology; Resources; Visualization; Writing – Original draft; Writing – Review & editing.

## DATA AVAILABILITY STATEMENT

All materials and analyses (including the R script), along with our preregistrations of methods and our expectations concerning the outcomes of our experiments can be found here: https://osf.io/7m86s/.
